# Development of an UV−Resistant Multilayer Film with Enhanced Compatibility between Carboxymethyl Cellulose and Polylactic Acid via Incorporation of Tannin and Ferric Chloride

**DOI:** 10.3390/molecules29122822

**Published:** 2024-06-13

**Authors:** Jian Xiao, Tingting Liu, Qiulu Chu, Chaoguang Yu, Yunlong Yin, Lei Xuan, Shufang Wu

**Affiliations:** 1 Jiangsu Co−Innovation Center of Efficient Processing and Utilization of Forest Resources, Nanjing Forestry University, 159 Longpan Road, Nanjing 210037, China; yydyt@njfu.edu.cn (J.X.); liutting0126@163.com (T.L.); chuqiulu@njfu.edu.cn (Q.C.); 2 College of Light Industry and Food Engineering, Nanjing Forestry University, 159 Longpan Road, Nanjing 210037, China; 3 Institute of Botany, Jiangsu Province and Chinese Academy of Sciences, Nanjing 210014, China; yuchaoguang168@cnbg.net (C.Y.); yinyl066@sina.com (Y.Y.); 13851991791@163.com (L.X.); 4 Jiangsu Key Laboratory for the Research and Utilization of Plant Resources, Nanjing 210014, China

**Keywords:** PLA, TA−Fe^3+^ complex, interface bonding, UV−resistant, wood protection

## Abstract

Carboxymethyl cellulose (CMC) and polylactic acid (PLA) are recognized for their environmental friendliness. By merging them into a composite film, packaging solutions can be designed with good performance. Nonetheless, the inherent interface disparity between CMC and PLA poses a challenge, and there may be layer separation issues. This study introduces a straightforward approach to mitigate this challenge by incorporating tannin acid and ferric chloride in the fabrication of the CMC−PLA. The interlayer compatibility was improved by the in situ formation of a cohesive interface. The resulting CMC/TA−PLA/Fe multilayer film, devoid of any layer separation, exhibits exceptional mechanical strength, with a tensile strength exceeding 70 MPa, a high contact angle of 105°, and superior thermal stability. Furthermore, the CMC/TA−PLA/Fe film demonstrates remarkable efficacy in blocking ultraviolet light, effectively minimizing the discoloration of various wood surfaces exposed to UV aging.

## 1. Introduction

Films play a pivotal role in various applications, such as food preservation, pharmaceutical encapsulation, and wood protection, owing to their superior qualities, like their lightweight nature, transparency, moisture resistance, and robust mechanical strength [1,2,3,4]. However, the reliance on petroleum−based materials for producing packaging films poses significant environmental and health risks due to their non−biodegradability. Consequently, there has been a growing interest in employing natural polymers like starch, chitosan, and gelatin for developing packaging films. These bio−based materials are not only safe and biodegradable but also imbued with additional functionalities such as antimicrobial, antioxidant, and UV protective properties [5,6,7].

Polylactic acid (PLA) is a biodegradable thermoplastic material that can be used in the production of fibers, coatings, and films for the food, textile, electronics, pharmaceutical, agricultural, and packaging industries [8,9]. Despite its advantages, PLA’s singular application often falls short of meeting the complex requirements of the market. An effective approach to this challenge involves integrating bioactive compounds into PLA to create multifunctional films. Tannin (TA), a widely available, renewable, and cost−effective polyphenol, possesses multiple bioactivities, such as UV resistance, antioxidation, and antimicrobial effects, offering additional functional benefits when combined with PLA [10]. Nevertheless, the integration of tannin into PLA is hindered by the latter’s low polarity and the high phenolic hydroxyl content of tannins, leading to suboptimal dispersion within PLA films. Efforts to enhance TA−PLA compatibility have included the modification of tannins through esterification or hydroxypropylation to diminish their polarity [11,12,13]. Additionally, Liao et al. achieved improved tensile properties and interfacial compatibility in PLA/TA composites using silane coupling agents [14]. However, while beneficial to compatibility, these modifications may compromise the composite film’s hydrophobicity and safety.

An alternative strategy to leverage tannin’s benefits in PLA films involves incorporating tannin into film−formable substances before creating multilayer films with PLA. Carboxymethyl cellulose (CMC), a cellulose derivative known for its excellent film−forming capability and compatibility with tannin, serves as an ideal candidate for this purpose [15]. Studies have indicated that CMC films infused with tannins exhibit exceptional UV resistance, and when combined with PLA, they can provide enhanced UV protective properties.

While the integration of CMC and PLA promises the development of multilayer films with enhanced properties, the dissimilar interface characteristics between the two materials often result in layer separation. Prior investigations have identified interfacial incompatibility leading to layer separation in multilayer films composed of PLA and various biomaterials, posing a significant challenge [16,17,18]. This concern extends to CMC and PLA composites, which are at risk of suffering from similar issues. To address this, several strategies have been explored: Muller utilized hot pressing to enhance the adhesion between starch and PLA layers [19]; Trinh and colleagues modified starch with maleic acid to bridge the interfacial gap between starch and PLA [20]; and Colli Pacheco et al. introduced amphiphilic paprika oleoresin as an intermediary layer to improve the interface between starch and PLA [21]. Similarly, tannins have been identified as potential enhancers of interfacial bonding, as exemplified by Hu et al.’s work in strengthening the bond between carbon fibers and epoxy resin through TA−Fe^3+^ complexes [22]. It can be seen that the construction of metal polyphenol complexes between multilayer films is beneficial for improving interface bonding. Fe^3+^ exhibits excellent biocompatibility and biodegradability. For instance, Zhou’s DDWP/TA/Fe film, constructed with tannin and Fe^3+^, and Wang’s CPL/Fe film, constructed with sodium lignosulfonate and Fe^3+^, both demonstrated remarkable biodegradability [23,24].

Wood is widely utilized in construction and interior design due to its low density, wide availability, strong mechanical properties, attractive texture, and biodegradability. However, when exposed to the outdoor environment, wood naturally ages as a result of ultraviolet light and moisture, leading to irreversible changes in its physical and chemical characteristics. While various methods have been developed to address UV and water−induced wood aging [25,26,27], most solutions involve applying surface coatings that can resist natural aging but may impact the wood’s subsequent sale and use. Another approach involves creating multifunctional packaging materials with UV shielding and hydrophobic properties to protect the wood during transportation and storage [2], thereby minimizing the direct impact on the wood itself.

In the present study, tannins and CMC were co−dissolved and Fe^3+^ was co−dissolved with PLA to fabricate a CMC/TA−PLA/Fe multilayer film, augmented with the TA−Fe^3+^ complex aimed at reinforcing the film’s structural integrity. The mechanical strength of this innovative multilayer film was thoroughly evaluated. Additionally, four types of wood commonly used in furniture were subjected to UV aging tests to assess the anti−UV aging efficacy of the CMC/TA−PLA/Fe film, demonstrating its potential to extend the lifespan of wood products exposed to UV radiation.

## 2. Results and Discussion

### 2.1. Characteristics of CMC−PLA Films

#### 2.1.1. Microscopic Morphology

**Figure 1:**
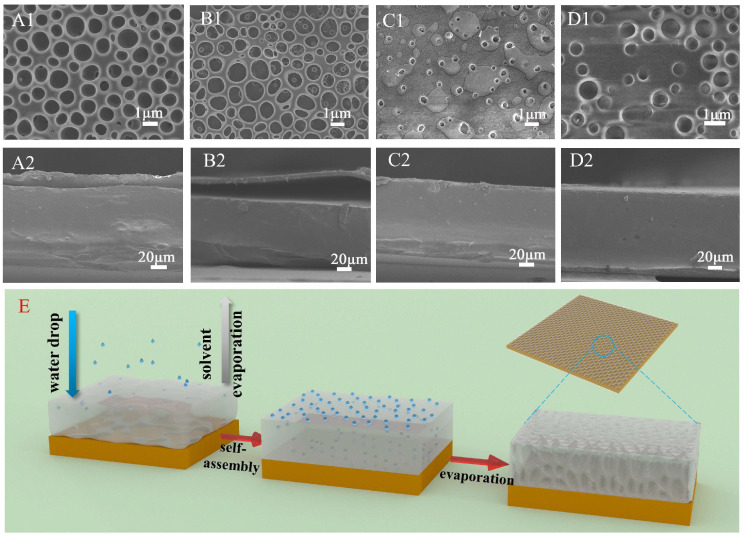
SEM images of the multilayer film CMC−PLA, CMC/TA−PLA, CMC−PLA/Fe, and CMC/TA−PLA/Fe (the surfaces were denoted as (**A1**−**D1**) and the cross−sections were denoted as (**A2**−**D2**)) and diagram of the self−assembly process of multilayer films (**E**). (CMC stands for carboxymethyl cellulose, TA stands for tannin, and PLA stands for polylactic acid).

The impact of incorporating tannin (TA) and/or ferric ions (Fe^3+^) on the interlayer cohesion of multilayer CMC−PLA films was examined via Scanning Electron Microscopy (SEM). SEM imagery, along with depictions of the self−assembly process within these films, is presented in Figure 1. Notably, all multilayer films exhibited a porous structure on their surfaces, a result of the breath figure (BF) method applied during the PLA layer’s formation [28]. This technique involved solvent evaporation, prompting water from the surrounding air to condense into droplets on the film surface, which subsequently evaporated post−PLA formation, leaving behind the observed porous architecture (Figure 1A1–D1) [29]. In order to further describe the state of pores on the surface of different samples, Image J software V1.8.0 was used to calculate the porosity and pore size of the film surface, and the results are shown in Table S1.

In samples without Fe^3+^, the surface porosity of CMC−PLA (Figure 1A1) and CMC/TA−PLA (Figure 1B1) films was 42.35% and 44.36%, respectively. The average pore area was 0.59 μm^2^ and 0.62 μm^2^, respectively. There was little difference between the two, indicating that the addition of tannin to the CMC layer alone did not significantly affect the surface morphology of the film. The addition of Fe^3+^ significantly reduced the porosity and pore area of the film surface, which was attributed to the fact that the chelation between Fe^3+^, tannin, and CMC reduced the distance between PLA molecules. Furthermore, there was about 2% FeCl_3_ contained in both samples, and the solvation of FeCl_3_ was thought to increase the surface energy of the droplets, consequently reducing their volume [30]. Compared to the CMC/TA−PLA/Fe (Figure 1D1) film, the pores on the surface of the CMC−PLA/Fe (Figure 1C1) film were smaller, with a porosity of only 3.12%, which was attributed to the limited cross−linking rate of CMC with Fe^3+^ compared to the rapid cross−linking of tannins with Fe^3+^. In the case of the CMC−PLA/Fe film, the limited rate of CMC binding to Fe^3+^ caused most of the FeCl_3_ to dissolve into the droplet, which resulted in a significant reduction in droplet volume and the deposition of FeCl_3_ around the pores. However, this issue was mitigated by the rapid cross−linking of tannin with FeCl_3_, which led to almost no FeCl_3_ particles on the surface of the CMC/TA−PLA/Fe film.

Examination of the cross−sectional SEM images of these films offers insights into layer adherence. Both CMC−PLA (Figure 1A2) and CMC/TA−PLA (Figure 1B2) exhibited visible layer separation, highlighting the inadequate binding between the hydrophobic PLA and hydrophilic CMC layers. In contrast, the introduction of Fe^3+^ mitigated this separation in CMC−PLA/Fe and CMC/TA−PLA/Fe films, underscoring Fe^3+^’s beneficial role in enhancing CMC and PLA layer cohesion. However, it is noteworthy that the absence of tannins in CMC−PLA/Fe results in significantly smaller PLA layer pores, potentially impacting the films’ surface characteristics.

#### 2.1.2. Hydrophobicity

**Figure 2:**
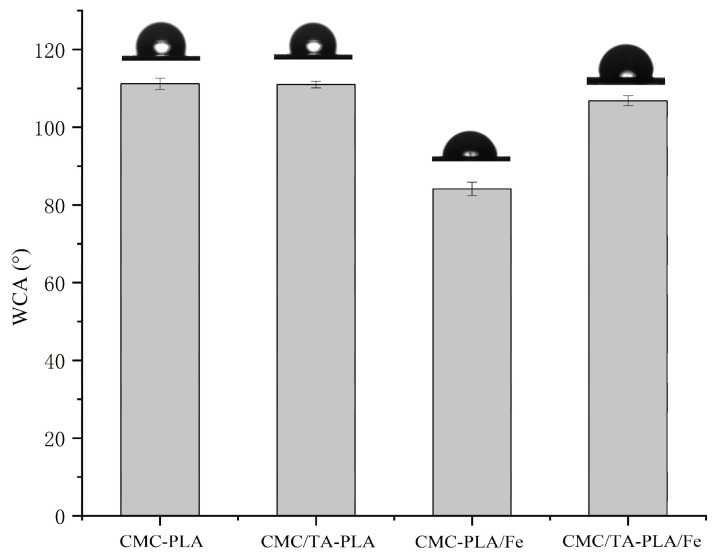
The WCA of multilayer.

The water contact angle (WCA) serves as a metric for assessing the hydrophobicity of the multilayer films, with findings illustrated in Figure 2. The CMC−PLA and CMC/TA−PLA films exhibited WCAs around 110°, indicative of a highly hydrophobic surface. This hydrophobicity is attributed to the porous structure of the PLA layer, which naturally repels water [31]. The CMC/TA−PLA/Fe film, however, registered a slightly lower WCA of 105°, a consequence of its diminished pore presence, leading to a relatively smoother surface [32], as corroborated by SEM observations (Figure 1). The absence of tannin further reduced the WCA to below 85°, significantly diminishing the film’s hydrophobic properties. This effect is largely due to the smaller and fewer pores, compounded by the deposition of FeCl_3_ around these pores, as depicted in Figure 1C1 [33].

#### 2.1.3. Mechanical Properties

**Figure 3:**
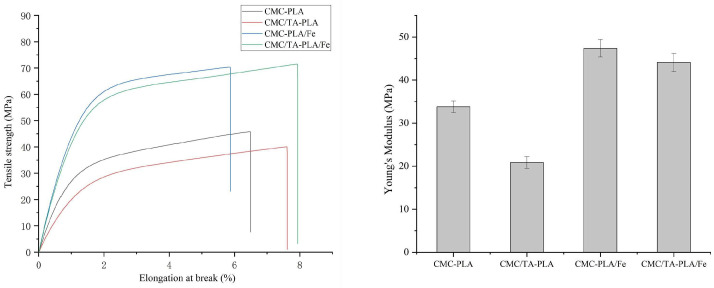
The mechanical properties of multilayer films.

The mechanical integrity of films is paramount for their practical application. Figure 3 delineates the mechanical strengths of the various multilayer films. It is worth mentioning that both CMC−PLA/Fe and CMC/TA−PLA/Fe films showed excellent tensile strength of about 70 MPa, which was higher than that of other biobased PLA multilayer films [20,34]. Interestingly, the CMC/TA−PLA/Fe film showed an increased elongation at the break of 8%, and its Young’s modulus decreased from 47.38 ± 2.03 MPa to 44.10 ± 2.11 MPa compared with the CMC−PLA/Fe film. The reason is that the addition of tannin increased the flexibility of CMC/TA−PLA/Fe film, while the deposition of FeCl3 increased the rigidity of CMC/TA−PLA/Fe film. In contrast, the films without Fe^3+^, namely CMC−PLA and CMC/TA−PLA, displayed reduced tensile strengths. The addition of TA further compromised the tensile strength of the CMC/TA−PLA film, albeit increasing its elongation at break to 7.61%. This decrease in tensile strength can be attributed to the disruption of stable hydrogen bonds within the CMC layer by tannin, leading to the formation of weaker bonds and increased molecular chain fluidity [35]. The introduction of Fe^3+^ to the CMC/TA−PLA multilayer films, however, enhanced both tensile strength and elongation at break. This improvement is attributed to stronger interlayer bonding, resulting in a more cohesive film structure capable of uniformly distributing tension across the film, thus better resisting external stress [36,37].

#### 2.1.4. UV Resistance Properties

**Figure 4:**
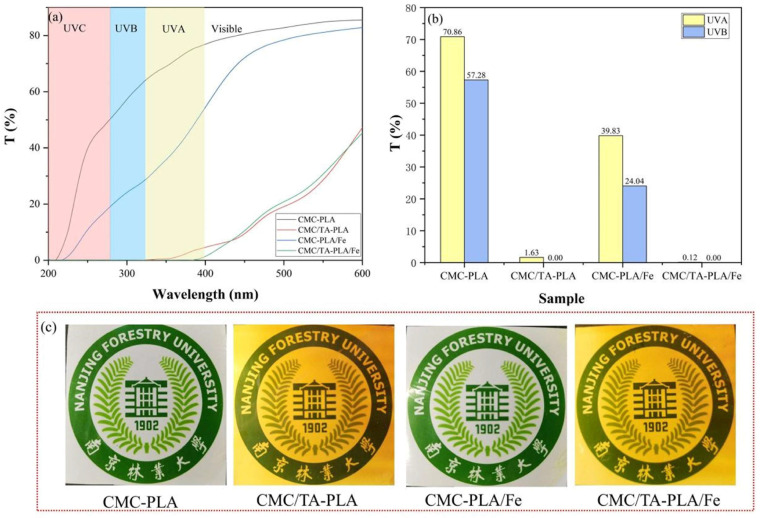
The transmittance of the multilayer films in the range of 200–600 nm (**a**); the average transmittance of UVA (Ultraviolet A) and UVB (Ultraviolet B) to the multilayer films (**b**); the digital photos of multilayer films (**c**).

The capability of films to shield against ultraviolet (UV) radiation is crucial, given the detrimental effects of UVA (320–400 nm) and UVB (280–320 nm) rays on material degradation and food spoilage, despite the near−complete absorption of UVC (200–280 nm) by the ozone layer [38,39,40]. The UV transmittance of the multilayer films across the 200–600 nm spectrum was evaluated, as depicted in Figure 4. The CMC−PLA film exhibited poor UV blocking, with average transmittance rates for UVA and UVB standing at 70.86% and 57.28%, respectively, closely mirroring the performance of a singular PLA layer film [41]. In contrast, the CMC−PLA/Fe film, despite lacking tannins, demonstrated a slight improvement in UV absorption, achieving up to 60% transmittance. Remarkably, films containing tannins, CMC/TA−PLA, and CMC/TA−PLA/Fe, showcased superior UV shielding, nearly nullifying UV penetration. This efficiency is attributed to tannin’s rich benzene ring structure [42]. The absence of tannin resulted in colorless and transparent films, whereas tannin incorporation rendered the films light brown and marginally less transparent (Figure 4c), a change that generally does not impede applications like wood protection.

#### 2.1.5. Thermal Stabilities

**Figure 5:**
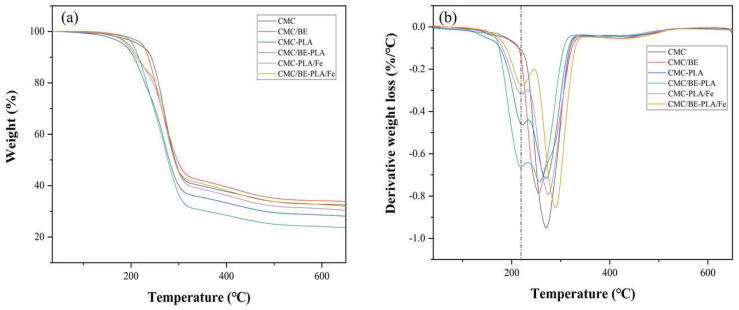
TG (**a**) and DTG (**b**) curves of multilayer films.

Thermogravimetric analysis (TGA) revealed that all multilayer films undergo two primary thermal degradation stages at 220 °C and between 240 and 340 °C (Figure 5), corresponding to the early decomposition of PLA and CMC, respectively [43,44]. The introduction of tannin led to a lower temperature degradation peak for CMC in CMC/TA−PLA films, indicating a diminution in thermal stability due to tannin disrupting CMC’s original network structure [45,46]. The subsequent addition of Fe^3+^ shifted the degradation peaks towards higher temperatures, with CMC/TA−PLA/Fe films peaking at 291 °C, surpassing the 274 °C peak of CMC−PLA. This shift is credited to the cross−linking between CMC and Fe^3+^ and the synergistic effect of tannin and Fe^3+^ [47,48]. Although the porous structure may affect the thermal conductivity of the material, the porous PLA layer had little effect on the thermal stability of the CMC layer due to its early degradation during heating.

Differential Scanning Calorimetry (DSC) determined the glass transition temperature (T_g_) and melting temperatures (T_m_) of the films (Table 1), showcasing a T_g_ of 66.91 °C for CMC−PLA and CMC/TA−PLA films, with T_m_ values of 161.72 °C and 162.32 °C, respectively, comparable to standalone PLA films [16]. The incorporation of Fe^3+^ elevated the T_g_ to 88.67 °C for CMC−PLA/Fe and 79.68 °C for CMC/TA−PLA/Fe, with no discernible melting points observed. The absence of T_m_ in the detected temperature range suggested that the introduction of Fe^3+^ increased the thermal stability of the films. The increase in Tg was attributed to the enhanced interlayer binding, which limited the flow of PLA molecular chains and might have affected the flexibility of the films [49,50]. In comparison to CMC/TA−PLA/Fe, the T_g_ of CMC−PLA/Fe films was higher, which was related to the increased rigidity caused by the deposition of FeCl_3_.

### 2.2. Mechanism of Improved the Interface Bonding between PLA and CMC by Chelation of Tannins and Fe^3+^

**Figure 6:**
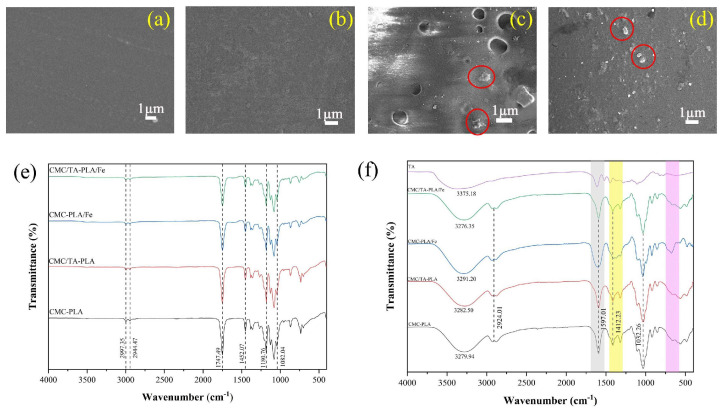
SEM images of the CMC layer CMC−PLA (**a**), CMC/TA−PLA (**b**), CMC−PLA/Fe (**c**), and CMC/TA−PLA/Fe (**d**) (the particles were marked in red circles); infrared spectrum of the surface (**e**) and CMC layer (**f**) of multilayer films.

The incorporation of tannin (TA) and ferric ions (Fe^3+^) has been pivotal in addressing the issue of layer separation in CMC−PLA films, in addition to enhancing their mechanical strength, thermal stability, and UV protection capabilities. It has been demonstrated in prior studies that Fe^3+^ can form complexes with tannin and CMC [51,52], suggesting that these complexes, once deposited on the CMC layer’s surface, act as a “bridge” to bolster the bond between PLA and CMC layers. This hypothesis was explored further by removing the PLA surface layer from the multilayer films for SEM analysis and FTIR measurements, with findings illustrated in Figure 6.

**Figure 7:**
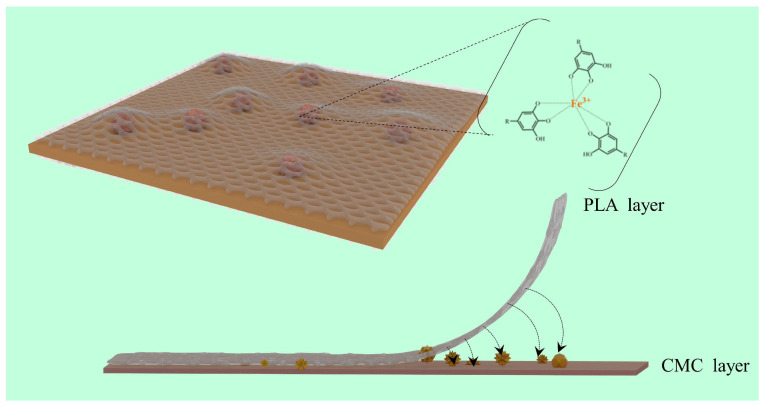
The mechanism diagram of the TA−Fe^3+^ composite complex enhanced the interface bonding between CMC and PLA of CMC/TA−PLA/Fe film.

SEM imagery reveals that the CMC layers of films without Fe^3+^ (Figure 6a,b) present a uniform and smooth surface akin to that of pure CMC films [53]. Conversely, for films incorporating Fe^3+^, granular protrusions were observed on the CMC layer’s surface (Figure 6c,d, as marked in the red circle). These granules, interspersed between the CMC and PLA layers, likely expand the interfacial contact surface, significantly enhancing the bond through increased mechanical interlocking [22]. This enhanced interlocking provides internal traction against external forces attempting to separate the layers (Figure 7).

Notably, defects were primarily observed on the CMC layer surface of the CMC−PLA/Fe film (Figure 6c). This suggests that, in the absence of TA, a substantial portion of FeCl_3_ integrates into the droplets formed during self−assembly, barring a minor fraction that quickly binds with CMC. These FeCl_3_−rich droplets fail to disperse evenly across the CMC surface or evaporate swiftly, leading to their prolonged presence on the CMC layer’s surface and the eventual emergence of defects [54]. This phenomenon may account for the lower elongation at break observed in the CMC−PLA/Fe film compared to the CMC/TA−PLA/Fe film without a corresponding increase in tensile strength.

The Fourier−transform infrared (FTIR) spectroscopy analysis of the CMC layer of films further substantiates the formation of TA−Fe^3+^ and CMC−Fe^3+^ complexes. The FTIR spectra for the CMC layers of CMC−PLA and CMC/TA−PLA, devoid of the PLA layer, bear resemblance owing to the akin infrared absorption characteristics of tannin and CMC [55,56,57]. A notable shift in the hydroxyl vibration peak from 3279.94 cm^−1^ in CMC−PLA to 3291.20 cm^−1^ in CMC−PLA/Fe suggests a stable coordination bond formation between Fe^3+^ and CMC, necessitating increased energy for molecular vibration [58]. Additionally, the broadening of the peak at 1579.01 cm^−1^ and the attenuation of peak intensity around 1412.23 cm^−1^ further corroborate the interaction between Fe^3+^ and CMC’s carboxyl groups [59]. The intensification of the peak at 680.85 cm^−1^, possibly related to Fe^3+^−CMC cross−linking (Figure 6f), aligns with findings from Li’s study [60].

For the CMC layer of CMC/TA−PLA/Fe film, the infrared spectra largely mirror those of both CMC/TA−PLA and CMC−PLA films, suggesting the predominant role of TA−Fe^3+^ binding in the film’s self−assembly. The swift association between Fe^3+^ and tannin curtails Fe^3+^’s binding affinity towards CMC, rendering the characteristic peak of the CMC−Fe^3+^ complex less pronounced. As to the PLA layer, the FTIR spectra of the multilayer films resemble that of pure PLA [61,62], implying that the enhanced interlayer adhesion between CMC and PLA is chiefly attributed to mechanical interlocking facilitated by TA−Fe^3+^ composite particles, without the formation of new covalent bonds. This insight highlights the sophisticated interplay of components within the multilayer films, emphasizing the role of mechanical and chemical interactions in achieving improved film properties.

### 2.3. Effect of the Film CMC/TA−PLA/Fe Prevent Wood Aging

The CMC/TA−PLA/Fe multilayer film, distinguished by its robust mechanical properties and superior UV shielding ability, is notably beneficial for various applications, including the preservation of wood. Wood’s distinct texture and mechanical strength have made it a favored material for outdoor construction and interior decoration. However, wood is susceptible to color changes induced by sunlight exposure, necessitating protective measures.

**Figure 8:**
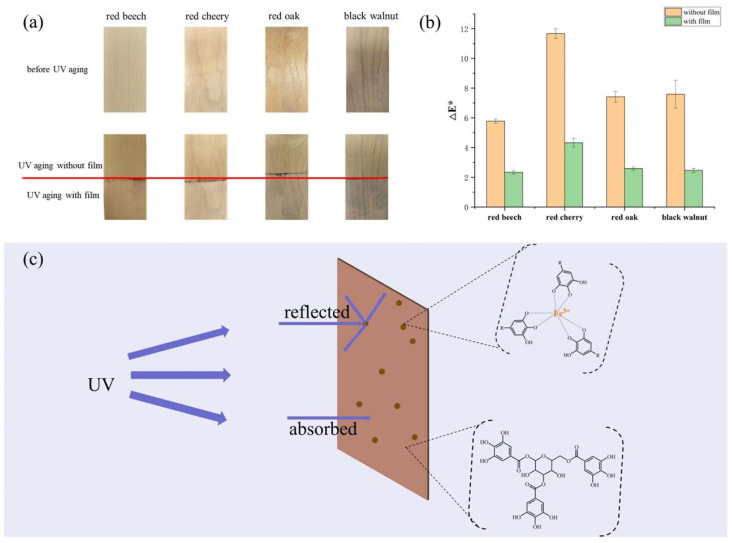
Digital photos of the boards before and after UV aging ((**a**), the covered part and the uncovered part were separated by red line), color changes (ΔE*) of boards before and after UV aging (**b**), and UV shielding diagram of CMC/TA−PLA/Fe (**c**).

This study evaluated the anti−aging efficacy of the CMC/TA−PLA/Fe film on four types of furniture wood: red beech, red cherry, red oak, and black walnut. Each wood sample was partially covered with the film for UV aging tests, and the outcomes, depicted in Figure 8, alongside CIE Lab values before and after UV exposure (Table S2), highlight the film’s protective capabilities. Post−UV aging, the uncovered portions of all wood samples demonstrated significant color alterations, with ΔE* values of 5.78, 11.67, 7.42, and 7.59, respectively (Figure 8b). Conversely, the sections shielded by the film displayed minimal changes. Relative to the unprotected wood, the ΔE* values decreased by approximately 70%, showcasing the film’s effectiveness in mitigating UV−induced color changes. The decrease in brightness (L*) and the increase in yellowness (b*) post−aging illustrate the wood’s darkening and yellowing, attributable to lignin oxidation [63,64].

The UV protective mechanism of the CMC/TA−PLA/Fe film, illustrated in Figure 8c, operates as follows: upon sunlight exposure, TA−Fe^3+^ composite particles within the film reflect a portion of UV light [65], while the majority of non−reflected UV radiation is absorbed by the film’s tannins, known for their abundant aromatic rings. As for visible light, a fraction is reflected, and the remainder, which reaches the wood, inflicts negligible damage [66].

## 3. Materials and Methods

### 3.1. Chemicals and Materials

Carboxymethyl cellulose sodium (CMC, viscosity 1000–1400 mPa·s) was sourced from Aladdin Reagent Co., Ltd., Shanghai, China, and utilized as supplied. Polylactic acid (PLA, grade 4032D) was procured from NatureWorks, Plymouth, MN, USA. Anhydrous ferric chloride (chemically pure, 400 mesh), glycerin (analytically pure), and tannin (analytically pure) were obtained from Sinopharm Chemical Reagent Co., Ltd., Shanghai, China, without further purification.

### 3.2. Preparation of CMC Film

**Figure 9:**
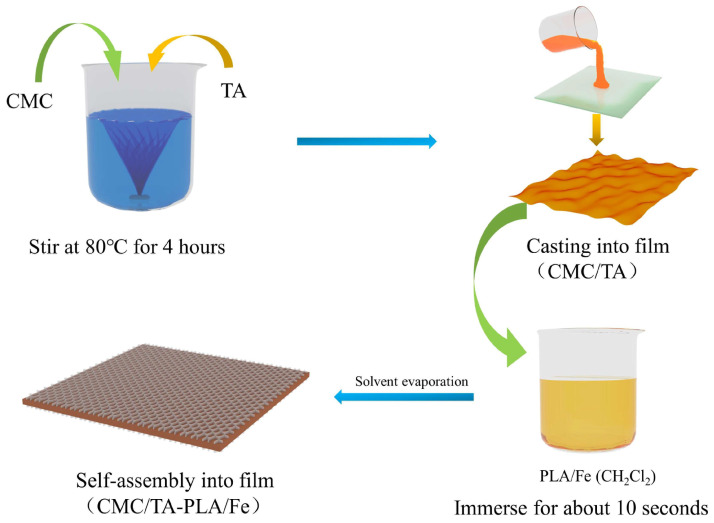
Preparation procedure of the film.

A film−forming solution was prepared by dissolving 4.0 g of CMC in distilled water to achieve a 2% (*w*/*v*) concentration. Subsequently, 0.2 g of tannin and 2.5 g of glycerol were incorporated into the solution. For comparative purposes, a control solution devoid of tannin was prepared following the same protocol. These solutions were then spread over a 20 cm × 20 cm glass plate and subjected to drying at 65 °C for 6 h. The resulting films were designated as CMC and CMC/TA, respectively. The entire film preparation procedure is depicted in Figure 9.

### 3.3. Preparation of Multilayer Films

For the preparation of multilayer films, PLA was dissolved in a dichloromethane (CH_2_Cl_2_) solution to form an 8% (*w*/*v*) concentration. Ferric chloride (FeCl_3_) was then added to this solution to achieve a concentration of 0.2 mol/L. An additional solution was also prepared without the inclusion of FeCl_3_. The CMC and CMC/TA films were each immersed in these solutions for 10 s. Following the evaporation of the solvent, four distinct film samples were produced: CMC−PLA, CMC/TA−PLA, CMC−PLA/Fe, and CMC/TA−PLA/Fe, as illustrated in Figure 9.

### 3.4. Characterization

#### 3.4.1. SEM Observation

The surface and cross−sectional morphologies of the films, including CMC−PLA, CMC/TA−PLA, CMC−PLA/Fe, and CMC/TA−PLA/Fe, were examined using Scanning Electron Microscopy (SEM) (Regulus 8100, Hitachi, Tokyo, Japan). Cross−sections were prepared by snap−freezing the samples in liquid nitrogen and fracturing them. The CMC layer’s surface morphology was also studied after carefully peeling the surfaces of the multilayer films. These analyses were conducted at an acceleration voltage of 5 kV, the samples were sprayed with gold by ion sputtering, and a secondary electronic detector was utilized for the detection process.

#### 3.4.2. Water Contact Angle (WCA) Measurement

The water contact angles of the films were determined using an Optical Contact Angle Tester (T200−Auto3 Plus, Biolin, Goteborg, Sweden). For this test, a 3 cm × 3 cm piece of film was placed on the instrument’s horizontal platform. Then, 3 μL of water droplets was carefully dropped onto the film surface, and the contact angle was measured from both sides. The static water contact angle (WCA) was measured 10 s after the water droplets were deposited on the sample surface. Each sample underwent five tests, and the average value was calculated.

#### 3.4.3. Mechanical Property Characteristic

Film samples were cut into strips measuring 50 mm × 10 mm for tensile testing. The tensile strength (in MPa) and elongation at break (in %) were measured under dry conditions using a universal tensile machine (AGS−X, Shimadzu, Kyoto, Japan) at a stretching speed of 10 mm/min.

#### 3.4.4. UV−Vis Transmittance Measurement

The optical transmittance of the films, with a thickness of 0.20 ± 0.02 mm, was assessed across the 200–600 nm wavelength range using a TU−1810 UV spectrophotometer (Persee Universal Instrument Co., Beijing, China).

#### 3.4.5. ATR−FTIR Analysis

The infrared spectra of the film samples were acquired in attenuated total reflection (ATR) mode using a Nicolet 560 infrared spectrometer (Nicolet, Madison, WI, USA), spanning a range from 4000 to 400 cm^−1^. This method was also applied to analyze the CMC layers’ spectra. Three areas of the sample surface were randomly selected for measurement, and each measurement point was scanned 16 times.

#### 3.4.6. TG Analysis

The thermal stability of the films was evaluated using thermogravimetric analysis (TGA209 F1, Netzsch, Bavaria, Germany). Samples weighing 5–8 mg were heated from 35 °C to 650 °C at a rate of 10 °C/min under a nitrogen atmosphere.

#### 3.4.7. DSC Analysis

Differential Scanning Calorimetry (DSC) was conducted using a DSC 214 (Netzsch, Selb, Germany) under a nitrogen atmosphere. Samples weighing 5–8 mg were placed in an aluminum crucible and subjected to a heating cycle from 35 °C to 180 °C at a rate of 10 °C/min, held at 180 °C for 10 minutes, then cooled to 35 °C at 10 °C/min. This cycle was repeated to determine the glass transition temperature (T_g_) and melting temperature (T_m_) using Advantage software V5.5.22.

### 3.5. Wood Aging Test

Boards of black walnut (*Juglans nigra* L.), red beech (*Fagus grandifolia*), red oak (*Quercus* spp.), and red cherry (*Prunus serotina*, Ehrh.), each measuring 5 cm (radial) × 10 cm (tangential) × 1 cm (longitudinal), were used for UV aging tests. The initial surface color of each board was recorded using a color measurement instrument (X−rite, Grandville, MI, USA) according to the CIELAB color system. The UV aging experiment referred to the method of Han et al. [2,67,68], and prior to aging, a CMC/TA−PLA/Fe film was wrapped around half of the wood in a single layer, and the packaging remained intact. Subsequently, the boards (cross−section) were exposed to 340 nm UV light for 72 h in a UV chamber set to 25 °C with a relative humidity of 65%. After exposure, the L*, a*, and b* values of both the wrapped and unwrapped sections of each board were measured. The color difference (ΔE*) was calculated using the formula:

$${\Delta E}^{*}=\sqrt{{\left({\Delta L}^{*}\right)}^{2}+{\left({\Delta a}^{*}\right)}^{2}+{\left({\Delta b}^{*}\right)}^{2}}$$

### 3.6. Statistical Analysis

Analysis of variance (ANOVA) was used to compare mean differences of the samples and the differences between the means were determined by the least significant difference (LSD) test at *p* ≤ 0.05.

## 4. Conclusions

In this study, a CMC/BE−PLA/Fe multilayer film was developed using the interfacial enhancement of tannin and ferric chloride. The film has excellent mechanical properties and exceptional UV shielding. SEM and infrared analysis demonstrated that the in situ formation of TA−Fe^3+^ particles enhanced the interface bonding between CMC and PLA. When tested for wood protection, the film was shown to reduce wood color change caused by ultraviolet light by 70%. This study not only serves as a valuable method for the development of sustainable packaging materials but also provides a viable strategy for enhancing the interface bonding of multilayer films.

## Figures and Tables

**Table 1:** The glass transition temperature (T_g_) and melting temperature (T_m_) of multilayer films.

Sample	T_g_ (°C)	T_m_ (°C)
CMC−PLA	66.91	161.72
CMC/TA−PLA	66.91	162.32
CMC−PLA/Fe	88.67	/
CMC/TA−PLA/Fe	79.68	/

## Data Availability

Data are contained within the article and Supplementary Materials.

## Supplementary Materials

The following supporting information can be downloaded at: https://www.mdpi.com/article/10.3390/molecules29122822/s1. Figure S1: DSC curve of multilayer films; Table S1: The surface porosity and pore area of the multilayer film; Table S2: The surface porosity and pore area of the multilayer film.
