# Supplementary material for: Development of an UV−Resistant Multilayer Film with Enhanced Compatibility between Carboxymethyl Cellulose and Polylactic Acid via Incorporation of Tannin and Ferric Chloride
Source: Molecules. 2024 Jun 13;29(12):2822. doi: 10.3390/molecules29122822 (PMC11206243; doi:10.3390/molecules29122822)
Supplement: Supplementary file 1 [file molecules-29-02822-s001.zip › molecules-3035552-supplementary.pdf]

Table S1. The surface porosity and pore area of the multilayer film

sample	pore area（μm^2）			porosity（%）
	Max.	Min.	Ave.	
CMC-PLA	1.56	0.01	0.59	42.35
CMC/TA-PLA	1.85	0.02	0.62	44.36
CMC-PLA/Fe	0.42	0.01	0.08	3.12
CMC/TA-PLA/Fe	0.72	0.02	0.25	16.86

Table S2. L*, a*, and b* values of the wood boards before and after UV aging

Samples		red beech	red cherry	red oak	black walnut
	untreated	70.84±0.06	61.35±0.79	72.69±0.55	53.99±0.60
L*	UV aging with film	67.33±0.29	50.68±0.50	66.49±0.84	47.31±0.11
	UV aging without film	69.47±0.25	57.22±0.60	70.34±0.47	51.56±0.43
	untreated	9.31±0.11	12.40±0.14	7.51±0.32	6.66±0.08
a*	UV aging with film	9.91±0.21	13.95±0.34	8.06±0.28	7.18±0.46
	UV aging without film	9.95±0.09	11.26±0.20	8.07±0.11	6.84±0.04
	untreated	19.48±0.15	20.82±0.07	19.61±0.54	13.27±0.00
b*	UV aging with film	24.00±0.43	25.26±0.08	23.11±0.33	16.80±0.58
	UV aging without film	21.23±0.06	20.53±0.07	18.73±0.30	13.02±0.25

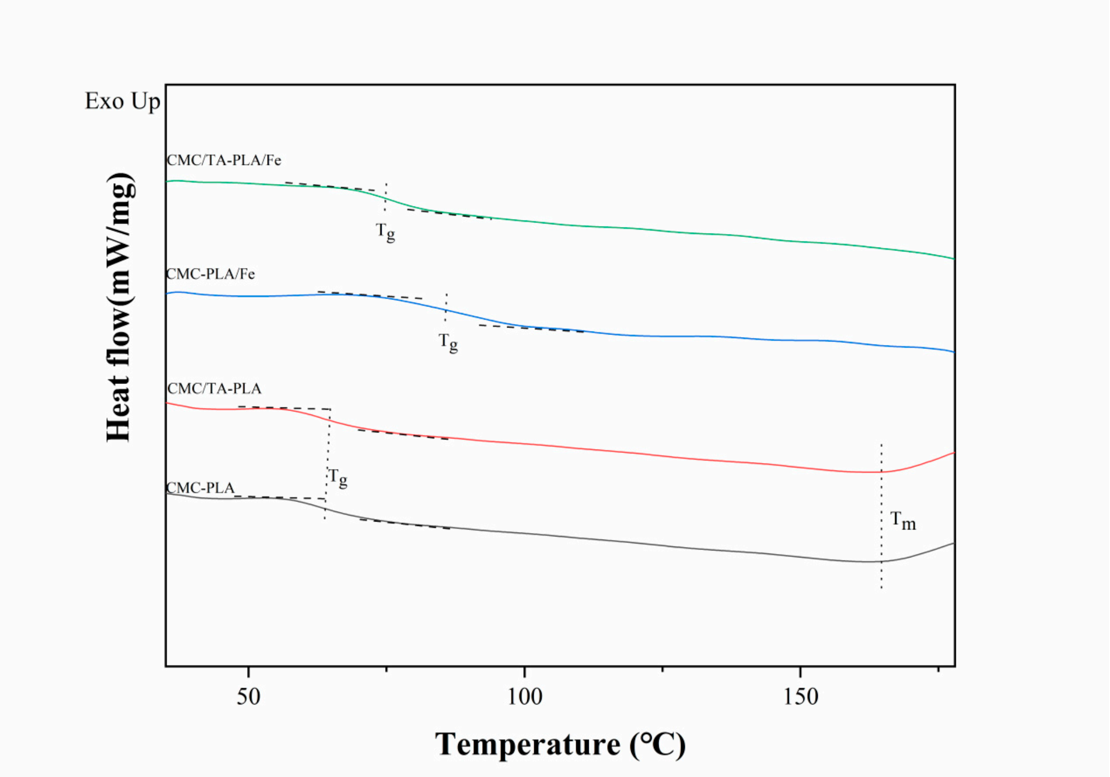

Figure S1. DSC curve of multilayer films
